# Estimating effects of health policy interventions using interrupted time-series analyses: a simulation study

**DOI:** 10.1186/s12874-022-01716-4

**Published:** 2022-08-31

**Authors:** Huan Jiang, Xinyang Feng, Shannon Lange, Alexander Tran, Jakob Manthey, Jürgen Rehm

**Affiliations:** 1 Institute for Mental Health Policy Research, Centre for Addiction and Mental Health (CAMH), 33 Ursula Franklin Street, Toronto, Ontario M5S 2S1 Canada; 2 Dalla Lana School of Public Health, University of Toronto, 155 College Street, 6th Floor, Toronto, Ontario M5T 3M7 Canada; 3 Campbell Family Mental Health Research Institute, CAMH, 250 College Street, Toronto, Ontario M5T 1R8 Canada; 4 Department of Psychiatry, University of Toronto, 250 College Street, 8th Floor, Toronto, Ontario M5T 1R8 Canada; 5 Department of Psychiatry and Psychotherapy, Center for Interdisciplinary Addiction Research (ZIS), University Medical Center Hamburg-Eppendorf (UKE), Martinistraße 52, 20246 Hamburg, Germany; 6 Institute of Clinical Psychology and Psychotherapy & Center for Clinical Epidemiology and Longitudinal Studies, Technische Universität Dresden, Chemnitzer Str. 46, 01187 Dresden, Germany; 7 Department of Psychiatry, Medical Faculty, University of Leipzig, Semmelweisstraße 10, 04103 Leipzig, Germany; 8 Institute of Medical Science (IMS), University of Toronto, Medical Sciences Building, 1 King’s College Circle, Room 2374, Toronto, Ontario M5S 1A8 Canada; 9 Department of International Health Projects, Institute for Leadership and Health Management, I.M. Sechenov First Moscow State Medical University, Trubetskaya str., 8, b. 2, Moscow, Russian Federation 119992

**Keywords:** Interrupted time-series, Segmented regression, GAMM, Quasi-experimental design, Policy evaluation, Simulation

## Abstract

**Background:**

A classic methodology used in evaluating the impact of health policy interventions is interrupted time-series (ITS) analysis, applying a quasi-experimental design that uses both pre- and post-policy data without randomization. In this paper, we took a simulation-based approach to estimating intervention effects under different assumptions.

**Methods:**

Each of the simulated mortality rates contained a linear time trend, seasonality, autoregressive, and moving-average terms. The simulations of the policy effects involved three scenarios: 1) immediate-level change only, 2) immediate-level and slope change, and 3) lagged-level and slope change. The estimated effects and biases of these effects were examined via three matched generalized additive mixed models, each of which used two different approaches: 1) effects based on estimated coefficients (*estimated* approach), and 2) effects based on predictions from models (*predicted* approach). The robustness of these two approaches was further investigated assuming misspecification of the models.

**Results:**

When one simulated dataset was analyzed with the matched model, the two analytical approaches produced similar estimates. However, when the models were misspecified, the number of deaths prevented, estimated using the *predicted* vs. *estimated* approaches, were very different, with the *predicted* approach yielding estimates closer to the real effect. The discrepancy was larger when the policy was applied early in the time-series.

**Conclusion:**

Even when the sample size appears to be large enough, one should still be cautious when conducting ITS analyses, since the power also depends on when in the series the intervention occurs. In addition, the intervention lagged effect needs to be fully considered at the study design stage (i.e., when developing the models).

**Supplementary Information:**

The online version contains supplementary material available at 10.1186/s12874-022-01716-4.

## Background

Given that the impact of regulatory and public policy interventions cannot usually be evaluated through traditional randomized controlled trial designs, well-selected, −designed, and -analyzed natural experiments are the method of choice when examining the effects of such enactments on a variety of outcomes [1–3]. A classic methodology for such evaluations is interrupted time-series (ITS) analysis, which is considered one of the quasi-experimental designs that uses both pre- and post-policy data without randomization and control series [4]. ITS is particularly well suited for interventions on the population level over a clearly defined time period [4, 5], and it has been used for the evaluation of various public health interventions with outcomes such as morbidity and mortality (e.g. [6]).

ITS needs to consider the order of data points and potential correlation of those points in time. For instance, many studies have reported seasonal variation in morbidity and mortality rates from various causes in different parts of the world. Taking mortality as an example, it has been observed that in many high-income countries of the Northern hemisphere all-cause mortality rates are the highest during the winter months and lowest in the summer months [7]. Thus, it is necessary to apply a statistical method that considers the effects of seasonality, trends, and other confounders when evaluating the intervention of interest.

Subsequent to estimating whether a policy intervention has had an effect over and above secular trends and chance [3], a quantification of the effect size in tangible units, such as the number of prevented cases/deaths, often is of great interest to various stakeholders as well as the wider public. As this information is key for future decisions aiming to maximize public health promotion and mitigate harms, the underlying estimation methods need to be as robust as possible. When using models to estimate the effects of a policy, it is also important to consider the extent to which the model accurately represents reality. Models can be used as an indicator of policy effects; however, there is always a risk that the modelled effect does not accurately describe the true effect on the outcome of interest. Thus, to accurately quantify a practical effect, it may be important to investigate the possibility of model misspecification.

So far, we have been somewhat abstract when talking about interventions and their effects. Therefore, let us consider, as an example, alcohol control interventions, such as increasing excise taxation to make alcohol less affordable or banning marketing (for a classification of such policies, see [8]) and their impact on mortality rates. Policy interventions can have an immediate effect, a lagged effect, or both, on mortality, depending on the type of policy and the cause of death of interest. An example of an outcome for which such policies would have an immediate effect would be deaths due to traffic injury [9]. However, taxation may have both an immediate and a lagged effect on liver cirrhosis mortality, as well as deaths from other chronic diseases (see [10] for an overview). As for banning alcohol marketing, most of the effects are expected to involve a lag-time–e.g., via young people receiving less exposure to alcohol advertisements, and thus the effect may take years to be fully realized. Therefore, for any public health intervention, assumptions about lag periods need to be made.

In ITS analyses, there are two main types of effects that describe the impact of a policy: first, there is the level change, which corresponds to the difference in the time point of interest and the predicted pre-intervention trend; and second, there is a slope change, which is a change in the time trend at one point in time [5]. To provide insight into how statistical methods performed under different effects, we took a simulation-based approach to estimate the potential effects of a simulated policy intervention on the original scale (e.g., death rate) and their uncertainty for ITS studies when the intervention impacts as 1) an immediate-level change only, 2) an immediate-level and slope change, or 3) a lagged-level and slope change. The intervention effect estimates and their accuracy were examined using two different methodological approaches, referred to as the ‘estimated’ and ‘predicted’ approach herein (for a description, see below). The robustness of these two approaches was further investigated assuming misspecification of the ITS models.

## Methods

To develop a basis for the simulations, we examined the ranges and variability of effect sizes from a previous time-series analyses of monthly age-standardized and sex-specific all-cause mortality rates (deaths per 100,000 adult population), for Lithuania from January 2000 to December 2019 [11, 12]. Model parameters adopted in the simulations were estimated using the monthly male mortality rate as a dependent variable. The seasonality of simulated data was assumed following a pattern represented by a cubic spline function, extracted from a fitted generalized additive mixed model (GAMM) using the monthly mortality rate among men. As indicated above, the simulated data included three different scenarios:

- Scenario 1: There was an immediate decrease, or level change, in the mortality rate after the policy intervention;
- Scenario 2: In addition to the immediate level change, the slope, i.e., the time trend of mortality rates, also changed after the intervention; and
- Scenario 3: We assumed that the full impact of the policy would take 2 years to be observed. In this scenario, the level change happened slowly across the 2 years post-intervention, as defined later, in addition to a slope change being observed after the two-year lag period.

### Simulated data

We defined effect size as the sum of expected level change plus the unit trend change, such as monthly change. Three effect sizes were used: 5, 10, and 15 deaths per 100,000, representing small, medium, and large reductions on monthly mortality rates before and after the intervention. In addition to differences in the effect size, the interventions were assumed to be different in their years of implementation during the study period. Specifically, interventions were applied in the beginning, middle, and later time of the study period, namely the 5th, 9th, and 13th year, respectively of the 18- year study period where the timings were chosen to reflect different study designs.

The conditional distribution of each observed ***y***_***t***_, for t = 1, …, n given the previous information of past observations ***y***_**1**_…, ***y***_***t*** − **1**_, and covariate vectors ***x***_**1**_, …, ***x***_***t***_, ***x***_***t***_ = {***x***_***t*1**_, …, ***x***_***tm***_} were assumed to follow the same Gaussian distribution. The simulated outcome ***y***_***t***_ was a random draw from a Gaussian distribution with mean value equal to the model expected value and variance. Each of the expected outcomes was determined by linear trend of time, seasonality, autoregressive (AR), and moving-average (MA) terms.

1 $${\boldsymbol\mu}_{\boldsymbol t}={\textstyle\sum_{\boldsymbol i=\mathbf1}^{\boldsymbol m}}{\mathbf X}_{\boldsymbol t\boldsymbol i}{\boldsymbol\beta}_{\boldsymbol i}+{\textstyle\sum_{\boldsymbol i=\mathbf1}^k}\boldsymbol s\left({\mathbf t}_{\boldsymbol i}\right)+{\textstyle\sum_{\boldsymbol j=\mathbf1}^{\boldsymbol p}}{\boldsymbol a}_{\boldsymbol j}\left({\boldsymbol y}_{\boldsymbol t-\boldsymbol j}-{\textstyle\sum_{\boldsymbol i=\mathbf1}^{\boldsymbol m}}{\mathbf X}_{\boldsymbol t-\boldsymbol j,\boldsymbol i}{\boldsymbol\beta}_{\boldsymbol i}\right)+{\textstyle\sum_{\boldsymbol j=\mathbf1}^{\boldsymbol q}}{\boldsymbol m}_{\boldsymbol j}\left({\boldsymbol y}_{\boldsymbol t-\boldsymbol j}-{\boldsymbol\mu}_{\boldsymbol t-\boldsymbol j}\right)$$

$${\boldsymbol{Y}}_{\boldsymbol{t}}\sim \mathbf{N}\ \left({\boldsymbol{\mu}}_{\boldsymbol{t}},{\boldsymbol{\sigma}}^{\mathbf{2}}\right)$$

Where $\sum_{\boldsymbol{i}=\mathbf{1}}^{\boldsymbol{k}}\boldsymbol{s}\left({\mathbf{t}}_{\boldsymbol{i}}\right)$ is smoothing cubic spline function for the monthly seasonal component with k = 12 being the number of knots, $\sum_{\boldsymbol{j}=\mathbf{1}}^{\boldsymbol{p}}{\boldsymbol{a}}_{\boldsymbol{j}}\left({\boldsymbol{y}}_{\boldsymbol{t}-\boldsymbol{j}}-\sum_{\boldsymbol{i}=\mathbf{1}}^{\boldsymbol{m}}{\mathbf{X}}_{\boldsymbol{t}-\boldsymbol{j},\boldsymbol{i}}{\boldsymbol{\beta}}_{\boldsymbol{i}}\right)$ are autoregressive terms of order p and $\sum_{\boldsymbol{j}=\mathbf{1}}^{\boldsymbol{q}}{\boldsymbol{m}}_{\boldsymbol{j}}\left({\boldsymbol{y}}_{\boldsymbol{t}-\boldsymbol{j}}-{\boldsymbol{\mu}}_{\boldsymbol{t}-\boldsymbol{j}}\right)$ are moving average terms of order q. Arbitrarily, ***p*** = ***q*** = **1** were chosen, suggested by the previous practices with the monthly mortality data of Lithuania [11, 12].

**Fig. 1:**
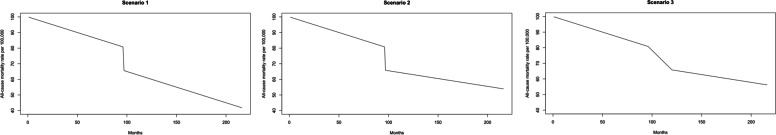
Illustration of the time trends of three scenarios

Figure 1 illustrates the three scenarios that were simulated. A detailed description about the value or distributions of variables can be found in the Additional file 1. For the first two scenarios, policy interventions were coded as abrupt permanent effects—i.e., assigning a value of 0 for all months preceding policy implementation and a value of 1 for all months following. For the third scenario where the policy implemented at time (T) needs 24 months to reach its full effect, the policy variable was represented by a step function (2):

2 $$X\_ Policy=\left\{\begin{array}{c}0\kern9.25em t<T\\ {}\frac{T-t}{24}\kern3em T<t<T+24\ \\ {}1\kern6.75em t>T+24\end{array}\right.$$

The ITS analyses were conducted on the simulated time series using generalized additive mixed models (GAMMs [4, 13];). Each simulated data set of three scenarios were analyzed using one of three GAMMs. The first GAMM (Model 1) assumes only an immediate level change, that is, the slope did not change after the intervention. *β*_1_ indicates the overall time trend and *β*_2_ is the post-intervention level change. The ‘trend’ variable refers to the linear time sequence and ‘level’ variable refers to the policy intervention.

3 $$\mathrm{Model}\ 1:\kern0.5em y={\beta}_0+{\beta}_1 trend+{\beta}_2\ {level}_t+{e}_t$$

The second GAMM used was as follows:

4 $$\mathrm{Model}\ 2:\kern0.5em y={\beta}_0+{\beta}_1 trend+{\beta}_2\ {level}_t+{\beta}_3\left[ trend\times {level}_t\right]+{e}_t$$

In this case, *β*_1_ represents the pre-intervention trend, *β*_2_ is the level change following the intervention and *β*_3_ shows the slope change following the intervention.

The third GAMM used is as follows:

5 $$\mathrm{Model}\ 3:\kern0.5em y={\beta}_0+{\beta}_1 trend+{\beta}_2\ {level}_t^{\prime }+{\beta}_3\left[ trend\times {level}_t^{\prime}\right]+{e}_t$$

${level}_t^{\prime }$ here was coded according to formula (2), considering the amount of time that the interventions would need to fully take effect (in our example, 24 months). The simulated datasets were analyzed with the respectively matched model, i.e., dataset describing Scenario 1 was analyzed with Model 1, Scenario 2 with Model 2, etc.

### Estimated policy impact

For each simulated cohort, the policy intervention effects were investigated with the matched GAMM model using two different methodological approaches, the: 1) ‘estimated’ and 2) ‘predicted’ method, as described below:

1. In the *estimated* approach, the intervention effect equals the beta weight from the ITS model (i.e., the classic ITS approach). As we are working with death rates in this example, the number of deaths averted by the intervention of interest in the 12 months following the intervention is derived by multiplying the beta-weight for the effect on the age-standardized mortality rate by the average population size for the 12 months following the intervention; and
2. In the *predicted* approach, there was a three-step process:

- Step 1: The data before the intervention is used to determine the optimal GAM for the series.
- Step 2: The GAM is used to forecast mortality rates for the 12 months following the intervention. For each month, the forecasted mortality rate and the 95% prediction intervals (PIs) were calculated assuming that the forecast errors were normally distributed.
- Step 3: The difference in mortality rates between observed and forecasted values after the intervention were multiplied by the corresponding population, resulting in an estimate of the absolute number of deaths being averted for each month. This estimate was considered to be the estimated number of averted deaths for the 12 months following the intervention, and the standard deviation was calculated by taking the square root of the combined variances of the predicted values, assuming independence between monthly data points. The 95% PI was further calculated assuming that the forecast errors were normally distributed.

In addition, sensitivity analyses to test the impact of model misspecification were conducted in which simulated data under Scenario 3 (a lagged effect) were analyzed with two simpler models: first, the data was analyzed using Model 1, which contains a time variable and policy, coded as a dummy variable: 0 before and 1 after the intervention; second, Model 2 was used and a slope change was added. For each of the three scenarios and each of the sensitivity analyses, a total of 1000 datasets were simulated and analyzed. For each of simulations, the estimated deaths prevented from the dataset were recorded. Under the Central Limit Theorem, the distribution of those estimates was approximately normal, and the 95% confidence interval (CI) was extracted over the sample of 1000 estimates. The coverage probability, defined as the proportion of iterations in which the true effect size was within the 95% CI surrounding the estimates, was then calculated.

## Results

### Scenario 1: immediate level change

When one simulated dataset was analyzed with the matched model, the two analytical approaches used produced similar estimates. In Scenario 1, for example, when the policy was applied in the 5th year of the study period with an immediate level effect only, deaths prevented were estimated to be 56, 122 and 179 when the effect size was − 5, − 10, and − 15, respectively, using the *estimated* approach, which roughly corresponds to the (monthly) effect size multiplied by 12 months, while the *predicted* approach resulted in 55, 118, and 176 deaths prevented, respectively (Table 1), with the results differing by less than 5%. However, the estimates with the *predicted* three-step method have much wider Cis—for the previous example, the 95% CIs were (1, 112), (71, 173) and (126, 233) using the *estimated* approach, compared to (− 43, 153), (24, 213) and (77, 275) using the *predicted* approach. This is as expected since the *predicted* method only utilized the data points before the intervention, decreasing the data size increased the width of CIs. When the policy was applied in the middle of the study period (i.e., the 9th year), mean estimates of death prevented were estimated to be 61, 121, and 181 when the effect size was − 5, − 10, and − 15, respectively, using the *estimated* approach, which is almost exactly the same as the *predicted* approach. However, again and as expected given the higher amount of underlying data, the 95% CIs were much wider for the *predicted* approach compared to the *estimated* approach. When the policy was applied in the later time of the study period—that is, the 13th year—the estimated number of deaths prevented was also the same with both approaches, except that the 95% CIs were, once again, slightly wider for the *predicted* approach. Specifically, they were (1117), (62, 176), and (120, 238) when the effect size was − 5, − 10, and − 15, respectively, with the *estimated* approach, and (− 15, 133), (44, 192), and (102, 256), respectively, with the *predicted* approach.

**Table 1:** Number of deaths prevented and their 95% confidence intervals (CI) for the three scenarios and their various assumptions

Year of implementation	Approach	Number of deaths prevented^a^	95% CI	Number of deaths prevented^a^	95% CI	Number of deaths prevented^a^	95% CI
Scenario 1		Effect size					
		−5		− 10		− 15	
	True value	60		120		180	
5	*Estimated*	56	(1, 112)	122	(71, 173)	179	(126, 233)
	*Predicted*	55	(−43, 153)	118	(24, 213)	176	(77, 275)
	True value	60		120		180	
9	*Estimated*	61	(0, 122)	121	(54, 188)	181	(118, 245)
	*Predicted*	61	(−18, 140)	120	(41, 200)	181	(101, 261)
	True value	50		120		180	
13	*Estimated*	59	(1, 117)	119	(62, 176)	179	(120, 238)
	*Predicted*	59	(−15, 133)	118	(44, 192)	179	(102, 256)
Scenario 2		Effect size					
		−5		−10		−15	
	True value	52		112		172	
5	*Estimated*	52	(7, 96)	112	(67, 156)	171	(127, 215)
	*Predicted*	50	(−50, 150)	111	(10, 212)	171	(71, 271)
	True value	52		112		172	
9	*Estimated*	52	(22, 82)	112	(80, 145)	172	(142, 202)
	*Predicted*	53	(−26, 131)	111	(30, 191)	172	(94, 251)
	True value	52		112		172	
13	*Estimated*	52	(21, 82)	113	(81, 144)	171	(141, 202)
	*Predicted*	51	(−23, 124)	111	(35, 187)	179	(94, 247)
Scenario 3		Effect size					
		−5		−10		−15	
	True value	1		17		33	
5	*Estimated*	0	(− 28, 28)	17	(−11, 45)	33	(4, 62)
	*Predicted*	−1	(− 93, 91)	16	(−75, 108)	32	(− 59, 124)
	True value	1		17		33	
9	*Estimated*	1	(−19, 20)	17	(−3, 37)	33	(13, 52)
	*Predicted*	0	(−74, 75)	15	(− 57, 88)	34	(−41, 108)
	True value	1		17		33	
13	*Estimated*	0	(−20, 21)	17	(−2, 35)	33	(14, 52)
	*Predicted*	0	(− 68, 67)	15	(−53, 83)	32	(−38, 102)

*CI* Confidence interval

^a^In the 12 months following the intervention

### Scenario 2: immediate level and slope change

For the second scenario with both an immediate-level change and a slope change, when the policy was applied in the 5th year of the study period, deaths prevented were estimated to be 52, 112, and 171 when the effect size was − 5, − 10, and − 15, respectively, using the *estimated* approach, compared to 50, 111, and 171 using the *predicted* approach. Similar to the first scenario, the 95% CIs were wider using the *predicted* approach. For example, they were (22, 82), (80,145), and (142, 202) when the effect size was − 5, − 10, and − 15, respectively, using the *estimated* approach when the policy was applied in the 9th year, compared to (− 26, 131), (30, 191), and (94, 251), respectively, using the *predicted* approach.

### Scenario 3: lagged level and slope change

Similar patterns can be observed in the third scenario, where a lagged policy effect was simulated. When the policy was applied in the 5th year of the study period, deaths prevented were estimated to be 0, 17, and 33 when the effect size was − 5, − 10, and − 15, respectively, using the *estimated* approach, compared to 0, 16 and 32, respectively, using the *predicted* approach. The *predicted* approach, again, had much wider 95% CIs. The lagged policy effect caused a much lower number of deaths averted by the intervention of interest in the 12 months following the intervention, compared to the previous two scenarios. The phenomenon was correctly captured with the matched models.

### Model misspecification

When the analytical models were misspecified, where the dataset simulated from Scenario 3 was analyzed using Model 1 and Model 2, the estimated number of deaths prevented were very different depending on whether the *predicted* or *estimated* approach was used. In almost all circumstance, the *estimated* approach overestimated the true effect. The discrepancy was the most notable when the policy was applied early in the study period. For instance, for Scenario 3 (lagged level and slope change) in which the policy was applied in the 5th year assuming a small effect size (reduction of 5 deaths per 100,000), we ran Model I and obtained 45 (95% CI: − 12, 101) deaths prevented using the *estimated* approach in contrast to − 1 (95% CI: − 93, 91) using the *predicted* approach (Table 2). The discrepancy between the two approaches becomes smaller when the policy is applied later in the study period. For example, when the policy was applied in the 13th year of the study, the *estimated* approach found − 7 (95% CI: − 63, 50) deaths were prevented and the *predicted* approach found 0 (95% CI: − 68, 67) deaths were prevented—the true number of deaths prevented was one. Since the ITS of the *predicted* approach only used data points from before the intervention, a later intervention means similar data points were used by the modeling steps in both approaches; therefore, the discrepancy between the approaches is likely reduced. When the same simulated data were analyzed using Model 2, with level and slope changes but without consideration of the two-year lag time, the discrepancy between the two approaches was less pronounced. For example, when the policy was applied in the 5th year of the study with an effect size of − 5, the true number of deaths prevented remained 1, and the *estimated* approach resulted in 13 (95% CI: − 67, 94) deaths prevented, while the *predicted* approach resulted in − 1 (95% CI: − 93, 91) deaths prevented. Given that the Model 2 also has a slope change, the parameters more closely resembled the effects seen in Scenario 3 (lagged effect, and slope change). Thus, the Model 2 produced more accurate effects when misspecified to Scenario 3.

**Table 2:** Sensitivity analyses: Number of deaths prevented after one year of policy implementation and their 95% confidence interval (CI) under Scenario 3 (lagged level and slope change) being analyzed with a misspecified model

Year of implementation	Approach	Number of deaths prevented^a^	95% CI	Number of deaths prevented^a^	95% CI	Number of deaths prevented^a^	95% CI
Scenario 3, using Model 1		Effect size					
		−5		−10		− 15	
	True values	1		17		33	
5	*Estimated*	45	(−12, 101)	85	(21, 148)	107	(9, 205)
	*Predicted*	−1	(−93, 91)	16	(−77, 108)	32	(− 60, 124)
	True values	1		17		33	
9	*Estimated*	28	(− 36, 91)	61	(−8, 130)	91	(11, 171)
	*Predicted*	1	(−73, 75)	16	(−59, 90)	34	(−41, 109)
	True values	1		17		33	
13	*Estimated*	−7	(−63, 50)	33	(−24, 91)	74	(14, 134)
	*Predicted*	0	(−68, 67)	15	(−55, 85)	32	(−38, 102)
Scenario 3, using Model 2		Effect size					
		−5		−10		−15	
	True values	1		17		33	
5	*Estimated*	13	(−67, 94)	58	(−27, 142)	85	(−20, 191)
	*Predicted*	−1	(−93, 91)	16	(−75, 108)	32	(−60, 124)
	True values	1		17		33	
9	*Estimated*	13	(− 49, 75)	51	(−16, 117)	83	(1, 164)
	*Predicted*	0	(−74, 75)	16	(−57, 89)	34	(−41, 109)
	True values	1		17		33	
13	*Estimated*	8	(−50, 66)	38	(−24, 99)	68	(3, 132)
	*Predicted*	0	(−68, 67)	15	(−55, 84)	32	(−38, 102)

*CI* Confidence interval

^a^In the 12 months following the intervention

When the dataset simulated from Scenario 1 was analyzed using Model 2, the estimated number of deaths prevented were very similar depending on whether the *predicted* or the *estimated* approach was used. Whereas, when it was analyzed using Model 3, the *predicted* method provided estimates closer to the true value than the *estimated* method. In other circumstances, e.g., when Scenario 2 was analyzed using Model 1 and Model 3, the *predicted* approach also produced estimates closer to the true values and performed better consistently (results are presented in the Additional file 1). It appears that the *predicted* approach was more robust under misspecification.

When the modelled policy effects (immediate level change, immediate level and slope change, or lagged level and slope change) were appropriately matched to the type of models used to analyze the simulated data, the practical effect (i.e., the number of deaths prevented) of the policy intervention can be very accurately determined. In fact, the coverage probability ranged between 85.6–90.7% (Table 3). Even when the models were misspecified, i.e., where the ITS model did not take the policy lagged effect into consideration, the coverage probabilities remained fairly stable. However, under the assumption that the policy had a lagged effect, the likelihood of the misspecified model estimates reflect the true effect size decreased significantly, especially when the policy was applied later in the study period. When Model 1 was applied to Scenario 3 and the policy was applied in the 13th year, the coverage probability was 30.7, 11.2, and 4.0% when the effect size was − 5, − 10, and − 15, respectively. On the other hand, when Model 2 without consideration of a lagged effect was applied to Scenario 3, the coverage probability was 61.5, 25.2, and 6.8% when the effect size was − 5, − 10, and − 15, respectively—i.e., slightly higher than Model 1. The coverage probabilities for the three scenarios with unmatched analysis depended largely on the year of implementation. For example, when Scenario 3 was analyzed using Model 1, the coverage probability was 0.858 when the policy was applied in the 5th year, compared to 0.307 when the policy was applied in the 13th year.

**Table 3:** Coverage probabilities for the three scenarios with matched and unmatched analysis

Effect size	Year of implementation	Coverage of 95% CI - matched analysis			Coverage of 95% CI - unmatched analysis	
		Scenario 1 with Model 1	Scenario 2 with Model 2	Scenario 3 with Model 3	Scenario 3 with Model 1	Scenario 3 with Model 2
−5	5	0.856	0.899	0.878	0.858	0.738
	9	0.885	0.884	0.875	0.804	0.652
	13	0.871	0.884	0.869	0.307	0.615
−10	5	0.892	0.896	0.879	0.715	0.623
	9	0.832	0.875	0.880	0.510	0.423
	13	0.882	0.873	0.889	0.112	0.252
−15	5	0.872	0.907	0.871	0.398	0.396
	9	0.882	0.897	0.874	0.256	0.210
	13	0.870	0.884	0.882	0.040	0.068

## Discussion

This simulation study began by fitting matched models to the simulated datasets and applying various circumstances—e.g., the point in time when the intervention happened within the series and different effect sizes of intervention, etc.—in order to gain a better understanding of how to best estimate the number of outcomes prevented following an effective population-level intervention. We found that when the model is correctly specified, both the *estimated* and *predicted* approaches produce similar estimates with a good likelihood of capturing the intervention effects, though the *estimated* approach often produces a much narrower 95% CIs than the *predicted* approach. However, when the model is misspecified**,** the *predicted* approach was found to produce estimates that were much closer to the true number of deaths prevented. As such, when one cannot determine which model is best for the data, the *predicted* approach should be used instead of the *estimated* approach.

Further, when the model is misspecified, the intervention effect might not be detected, especially when the policy occurs later in the study period, and it takes time for its full effect to be observed. Even when the number of time points post-implementation appears large enough (for criteria see [4]), the coverage probability was markedly reduced. This suggests that studies with unbalanced designs (in terms of time points before and after the intervention) and fewer data points after the intervention tend to have less probability of identifying a true effect when compared to studies with an equal number of time points before and after the intervention. In such cases, one should be cautious when conducting ITS analyses even when the sample size appears large enough according to common practice [4], since the power also depends on when the intervention occurs within the series [14]. Additionally, the lag effect of an intervention on a particular outcome of interest needs to be considered carefully at the study design stage (i.e., when developing the models). However, lag specifications are currently not well addressed in the literature [10].

There are a few limitations to the current study. First, we assumed a linear progression of the lagged effects (i.e., a gradual increase with equal increments over time). However, depending on the outcome of interest, it may take on a different form of progression and be subject to the law of diminishing marginal value (i.e., there comes a point when there is a lessening of impact). Secondly, the parameters used in the simulation study are based on the estimates abstracted from an analysis of Lithuania mortality data [12], and from our experience working with this or similar datasets [15, 16]. Therefore, the parameters used might not reflect all possibilities. For example, the seasonality might have different characteristics and the autoregressive component of GAMM model might have different signals for different studies.

In conclusion, aside from such parameter considerations, one needs to be cautious when estimating the number of deaths prevented when 1) the interventions occur later rather than in the middle of the time-series; 2) there is the possibility of a lag between the time a health policy is implemented and the shift in the outcome of interest; and 3) when there is a chance of model misspecification. Unfortunately, these conditions describe the majority of practical examples in applied ITS [17]. Improved model specification techniques via pooling knowledge from other studies on similar interventions, better knowledge on shape of temporal impact of interventions, and more simulation studies to better understand where most biases may be generated are key for improving this important application field for public policy.

## Supplementary Information

**Additional file 1: Appendix Table A1.** List of model coefficients. **Appendix Table A2.** Sensitivity analyses: Number of deaths prevented after one year of policy implementation and their 95% confidence interval (CI) under Scenario 1 (lagged level and slope change) being analyzed with a misspecified model. **Appendix Table A3.** Sensitivity analyses: Number of deaths prevented after one year of policy implementation and their 95% confidence interval (CI) under Scenario 2 (lagged level and slope change) being analyzed with a misspecified model.

## Data Availability

The datasets generated and/or analysed during the current study are not publicly available but are available from the corresponding author on reasonable request.

## Abbreviations

ARIMA: Autoregressive integrated moving average
GAMM: General additive mixed model
PI: Prediction Interval
CI: Confidence Interval
ITS: Interrupted time series
WHO: World Health Organization
