# Supplementary material for: Estimating effects of health policy interventions using interrupted time-series analyses: a simulation study
Source: BMC Med Res Methodol. 2022 Aug 31;22:235. doi: 10.1186/s12874-022-01716-4 (PMC9429656; doi:10.1186/s12874-022-01716-4)
Supplement: Supplementary file 1 — Additional file 1: Appendix Table A1. List of model coefficients. Appendix Table A2. Sensitivity analyses: Number of deaths prevented after one year of policy implementation and their 95% confidence interval (CI) under Scenario 1 (lagged level and slope change) being analyzed with a misspecified model. Appendix Table A3. Sensitivity analyses: Number of deaths prevented after one year of policy implementation and their 95% confidence interval (CI) under Scenario 2 (lagged level and slope change) being analyzed with a misspecified model. [file 12874_2022_1716_MOESM1_ESM.docx]

**Appendix**

**Table A1.** List of model coefficients

|  | Model 1 | Model 2 &3 |
| --- | --- | --- |
| $\beta_{0}$ | 100 | 100 |
| $\beta_{1}$ | -0.2 | -0.2 |
| $\beta_{2}$ | -5, -10, -15 | -5, -10, -15 |
| $\beta_{3}$ | - | 0.1 |
| $a$ | 0.6 | 0.6 |
| $m$ | -0.8 | -0.8 |

*Note.* The intercept term $\beta_{0}$ represents the mortality rate in the beginning of the study. The slope parameter $\beta_{1}$ describes the linear trend of time. The policy effect coefficient $\beta_{2}$ is the post-intervention level change in mortality rate. The interaction parameter $\beta_{3}$ represents the interaction effect between policy and time. The time-series components were generated form the stationary Gaussian autoregressive moving average distribution with the coefficient of autoregressive terms being 0.6, and the coefficient of moving average terms being -0.8. The error variance parameter of the time-series is assumed to be 25.

**Table A2.** Sensitivity analyses: Number of deaths prevented after one year of policy implementation and their 95% confidence interval (CI) under Scenario 1 (lagged level and slope change) being analyzed with a misspecified model

| Scenario 1, using Model 2 | | Effect size | | | | | |
| --- | --- | --- | --- | --- | --- | --- | --- |
|  |  | -5 | | -10 | | -15 | |
| Year of implementation | Method | Deaths prevented^a^ | 95% CI | Deaths prevented^a^ | 95% CI | Deaths prevented^a^ | 95% CI |
|  | True values | 60 |  | 120 |  | 180 |  |
| 5 | *Estimated* | 57 | (-25, 139) | 121 | (40, 202) | 184 | (108, 260) |
|  | *Predicted* | 60 | (-38, 157) | 119 | (25, 214) | 184 | (92, 276) |
|  | True values | 60 |  | 120 |  | 180 | 60 |
| 9 | *Estimated* | 62 | (-2, 125) | 119 | (54, 184) | 180 | (114, 247) |
|  | *Predicted* | 61 | (-17, 139) | 117 | (36, 198) | 181 | (99, 263) |
|  | True values | 60 |  | 120 |  | 180 | 60 |
| 13 | *Estimated* | 60 | (-6, 125) | 120 | (57, 182) | 181 | (117, 245) |
|  | *Predicted* | 59 | (-15, 134) | 119 | (44, 195) | 181 | (104, 259) |
| Scenario 1, using Model 3 | | Effect size | | | | | |
|  |  | -5 | | -10 | | -15 | |
| Year of implementation | Method | Deaths prevented^a^ | 95% CI | Deaths prevented^a^ | 95% CI | Deaths prevented^a^ | 95% CI |
|  | True values | 60 |  | 120 |  | 180 |  |
| 5 | *Estimated* | 7 | (-19, 32) | 15 | (-9, 39) | 21 | (-5, 46) |
|  | *Predicted* | 60 | (-38, 157) | 119 | (25, 214) | 184 | (92, 276) |
|  | True values | 60 |  | 120 |  | 180 |  |
| 9 | *Estimated* | 13 | (-5, 31) | 25 | (6, 44) | 36 | (15, 57) |
|  | *Predicted* | 61 | (-16, 138) | 117 | (36, 198) | 181 | (99, 264) |
|  | True values | 60 |  | 120 |  | 180 |  |
| 13 | *Estimated* | 16 | (-4, 36) | 31 | (12, 50) | 43 | (21, 65) |
|  | *Predicted* | 59 | (-15, 134) | 119 | (44, 195) | 180 | (105, 256) |

**Table A3.** Sensitivity analyses: Number of deaths prevented after one year of policy implementation and their 95% confidence interval (CI) under Scenario 2 (lagged level and slope change) being analyzed with a misspecified model

| Scenario 2, using Model 1 | | Effect size | | | | | |
| --- | --- | --- | --- | --- | --- | --- | --- |
|  |  | -5 | | -10 | | -15 | |
| Year of implementation | Method | Deaths prevented^a^ | 95% CI | Deaths prevented^a^ | 95% CI | Deathsprevented^a^ | 95% CI |
|  | True values | 52 |  | 112 |  | 172 |  |
| 5 | *Estimated* | 59 | (22, 97) | 120 | (81, 158) | 179 | (142, 217) |
|  | *Predicted* | 50 | (-50, 150) | 111 | (10, 212) | 171 | (71, 271) |
|  | True values | 52 |  | 112 |  | 172 |  |
| 9 | *Estimated* | 60 | (31, 90) | 120 | (89, 151) | 180 | (150, 209) |
|  | *Predicted* | 53 | (-26, 131) | 111 | (30, 192) | 172 | (94, 251) |
|  | True values | 52 |  | 112 |  | 172 |  |
| 13 | *Estimated* | 59 | (27, 92) | 120 | (86, 154) | 179 | (147, 212) |
|  | *Predicted* | 51 | (-23, 124) | 111 | (35, 188) | 170 | (94, 247) |
| Scenario 2, using Model 3 | | Effect size | | | | | |
|  |  | -5 | | -10 | | -15 | |
| Year of implementation | Method | Deaths prevented^a^ | 95% CI | Deaths prevented^a^ | 95% CI | Deaths prevented^a^ | 95% CI |
|  | True values | 52 |  | 112 |  | 172 |  |
| 5 | *Estimated* | 0 | (-17, 18) | 16 | (-2, 34) | 33 | (16, 50) |
|  | *Predicted* | 50 | (-50, 150) | 110 | (6, 214) | 171 | (69, 272) |
|  | True values | 52 |  | 112 |  | 172 |  |
| 9 | *Estimated* | 1 | (-9, 10) | 17 | (6, 27) | 33 | (23, 43) |
|  | *Predicted* | 52 | (-26, 131) | 109 | (28, 190) | 173 | (93, 253) |
|  | True values | 52 |  | 112 |  | 172 |  |
| 13 | *Estimated* | 1 | (-9, 10) | 17 | (7, 27) | 33 | (23, 42) |
|  | *Predicted* | 51 | (-23, 125) | 111 | (35, 187) | 171 | (95, 247) |
